# Spatio-Temporal Credit Assignment in Neuronal Population Learning

**DOI:** 10.1371/journal.pcbi.1002092

**Published:** 2011-06-30

**Authors:** Johannes Friedrich, Robert Urbanczik, Walter Senn

**Affiliations:** Department of Physiology, University of Bern, Bern, Switzerland; École Normale Supérieure, College de France, CNRS, France

## Abstract

In learning from trial and error, animals need to relate behavioral decisions to environmental reinforcement even though it may be difficult to assign credit to a particular decision when outcomes are uncertain or subject to delays. When considering the biophysical basis of learning, the credit-assignment problem is compounded because the behavioral decisions themselves result from the spatio-temporal aggregation of many synaptic releases. We present a model of plasticity induction for reinforcement learning in a population of leaky integrate and fire neurons which is based on a cascade of synaptic memory traces. Each synaptic cascade correlates presynaptic input first with postsynaptic events, next with the behavioral decisions and finally with external reinforcement. For operant conditioning, learning succeeds even when reinforcement is delivered with a delay so large that temporal contiguity between decision and pertinent reward is lost due to intervening decisions which are themselves subject to delayed reinforcement. This shows that the model provides a viable mechanism for temporal credit assignment. Further, learning speeds up with increasing population size, so the plasticity cascade simultaneously addresses the spatial problem of assigning credit to synapses in different population neurons. Simulations on other tasks, such as sequential decision making, serve to contrast the performance of the proposed scheme to that of temporal difference-based learning. We argue that, due to their comparative robustness, synaptic plasticity cascades are attractive basic models of reinforcement learning in the brain.

## Author Summary

The key mechanisms supporting memory and learning in the brain rely on changing the strength of synapses which control the transmission of information between neurons. But how are appropriate changes determined when animals learn from trial and error? Information on success or failure is likely signaled to synapses by neurotransmitters like dopamine. But interpreting this reward signal is difficult because the number of synaptic transmissions occurring during behavioral decision making is huge and each transmission may have contributed differently to the decision, or perhaps not at all. Extrapolating from experimental evidence on synaptic plasticity, we suggest a computational model where each synapse collects information about its contributions to the decision process by means of a cascade of transient memory traces. The final trace then remodulates the reward signal when the persistent change of the synaptic strength is triggered. Simulation results show that with the suggested synaptic plasticity rule a simple neural network can learn even difficult tasks by trial and error, e.g., when the decision - reward sequence is scrambled due to large delays in reward delivery.

## Introduction

Learning from reinforcement involves widely differing spatial and temporal scales both within the behavioral decision making process itself as well as when relating decisions to outcomes. Since they are adaptive, synapses may be viewed as the elementary decision making entities in the brain. But the presynaptic input of any single synapse will contain only very limited information about the task and, further, the millisecond duration of a synaptic release is much shorter than behaviorally relevant time scales. The behavioral decision results from a spatio-temporal aggregation of synaptic releases which is highly non-linear due to e.g. thresholding in the generation of action potentials. Hence the relationship between any single synaptic release and the behavioral decision is not only tenuous but also non-linear.

In relating behavioral decisions to rewarding or unrewarding outcomes, problems arise which are analogous to the ones encountered when relating synaptic releases to decisions. In the “spatial” domain: The state of the world is only partially observable, and hence, what appears to be one and the same decision may sometimes be rewarded and sometimes not. Also, in social interactions, reward may depend on the decisions of other players. In the temporal domain: Whether a decision was appropriate or not may not be immediately obvious and reward may even change with time. Proverbially, short term gain may lead to long term pain (and vice versa).

Hence the spatio-temporal credit assignment problem arises: How can a synapse adapt given that reward delivery is delayed and also depends on the releases of many other synapses as well as on external factors? As one basic mechanism for addressing the temporal problem, theories of reinforcement learning use the eligibility trace, a quantity, decaying exponentially in time, which memorizes the elementary decision up to the time when information about reward becomes available to trigger the persistent adaptive change [1]. Here we point out that a cascade of such synaptic memory traces can in fact provide an integrated solution to the spatio-temporal credit assignment problem by remodulating the presynaptic signal in view of information arising at different stages of the behavioral decision making.

Evidence for synaptic eligibility traces comes from experiments on spike timing dependent plasticity (STDP) where a synaptic release leads to longterm potentiation (LTP) if the neuron emits an action potential shortly thereafter [2], [3]. Importantly, the length of the LTP-induction time window (some 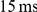) is on the order of the membrane time constant (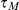), i.e. it reflects the time during which the synaptic release has influence on somatic action potential generation. The release itself lasts only for some 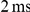, so this form of LTP is most easily accounted for by assuming a local synaptic quantity 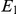 providing, just like an eligibility trace, a memory of the release which decays with time constant 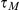. When an action potential is generated, 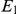 is read-out to determine a quantity 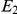 which, in the simplest interpretation of the STDP findings, gives the change (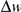) of the synaptic strength [4]. Simply equating 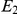 with 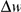, however, may be hasty because many repeated pre/post pairings are required in the STDP-protocol to induce a noticeable change. So it seems more reasonable to view 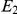 as a second synaptic eligibility trace, keeping a running record of recent pre/post pairings to modulate synaptic strength, perhaps even in a non-linear manner.

**Figure 1:**
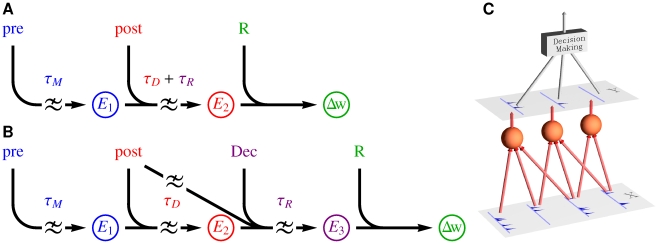
Plasticity cascades and decision making. (A) Synaptic plasticity cascades for reinforcement learning in the single neuron approach and (B) in the proposed population level approach. The meaning of the symbols is the following. 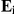: synaptic eligibility traces, 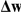: change in synaptic strength, **pre**: synaptic input, **post**: feedback from the postsynaptic neuron, 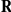: external reward feedback, **Dec**: feedback about the behavioral decision. The symbol denotes low pass filtering with the time constant 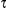 given next to the symbol. (C) Sketch of the studied population model for reinforcement learning: A stimulus 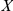 is read by a population of neurons yielding a spatio-temporal activity pattern 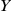 which depends on the synaptic strength of the neurons. A decision making circuitry transforms the population response 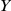 into a behavioral decision. The synaptic strength of the neurons should adapt so that population responses lead to behavioral decisions which maximize an external reward signal.

As has been widely noted [5]–[11], one can connect the STDP-findings with reinforcement learning by assuming that the transcription of the second eligibility trace 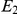 into the synaptic change 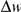 is modulated by neurotransmitters like dopamine which provide feedback about external reward (Fig. 1A). Such plasticity rules address the spatial credit assignment problem for synapses sharing a postsynaptic neuron since 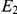 captures the relevant correlations between a given synaptic release and the releases of other synapses when they contribute to postsynaptic firing in the neuron. But 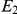 does not take into account the interaction in decision making between synapses which have different postsynaptic neurons. For temporal credit assignment, the memory length of 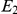 must correspond to the delay between a synaptic release and the delivery of pertinent reward feedback. This delay consists of the time 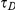 needed to reach a behavioral decision and the time 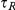 for this decision to be rewarded. A value on the order of 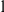 s seems reasonable for 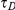, but 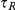 can easily be much longer, as in a game where multiple decisions are needed to reach a rewarding state. In this case, 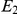 simply averages pre/post pairing over multiple decisions even if the firing of the particular neuron was important only for some of the decisions.

Here we propose extending the eligibility trace cascade by a further trace 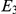 which takes into account the behavioral decision making process (Fig. 1B). Now the time constant of 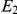 is simply 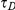, since 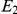 only needs to capture pre/post pairings upto the time when a decision is reached. The decision triggers a transcription of 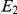 into 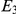 which is modulated by a feedback signal from the decision making circuitry and a signal derived from the firings of the postsynaptic neuron during the decision period. So while 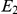 only captures the pre/post correlations, 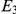 additionally captures the post/decision correlations. The time constant of 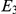 is 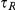, and when reward feedback does become available, the reward together with 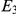 determines the synaptic change 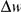.

In Text S1 we show that, for a population of spiking neurons feeding into a decision making circuitry (Fig. 1C), such a synaptic cascade can be mathematically derived by calculating the gradient of the expected reward. The resulting gradient ascent rule, however, has a few biologically undesirable aspects. For instance, it requires that 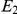 averages pre/post correlations over each decision period. Synapses, however, are unlikely to know when decision periods start and end. For biological realism, we present a modified rule in the main text, where e.g. the averaging over the decision period is replaced by low pass filtering. Learning in a population of spiking neurons using this synaptic plasticity rule is illustrated by simulation results. These show that learning speeds up with increasing population size and that learning speed degrades gracefully when the delay period between decision and reinforcement is increased. In particular, perfect performance is approached even when in the delay period the network has to make further decisions which themselves give rise to delayed reinforcement.

Eligibility traces memorize information about the decision making upto the time when reinforcement becomes available. In contrast, temporal difference (TD) learning, the other basic approach for temporal credit assignment in reinforcement learning, back-propagates reward to the time of the decision. For this, TD-learning estimates the value of states, or state-decision pairs, where, in the simplest case, a state corresponds to a stimulus. The value itself is the (discounted) expected future reward when being in the state, or when making a particular decision in the state. The value can then serve as an immediately available surrogate for the delayed reward signal. During Pavlovian learning, a backward shift in time is observed for the appetitive reaction from the delayed unconditioned stimulus to the conditioned stimulus, and the shift is found as well in the activity of midbrain dopaminergic neurons. The backward shift also occurs in the value estimation error computed by a TD-algorithm modeling the conditioning task, when a state of the algorithm corresponds to the time elapsed since the presentation of the conditioning stimulus [12]. Further to this observation, there has been a surge of interest in modeling dopaminergic activity in terms of TD-learning concepts, as reviewed in [13].

Temporal difference algorithms are based on the assumption that the information available for decision making is rich enough to make the learning problem Markovian. This means that the future is independent of past events, given the current state accessible to the TD-learner. In contrast, eligibility trace based approaches such as our population learning do not require such a completeness of available information. Hence, we present simulation results comparing the performance of the proposed approach to that of TD-learning on tasks, where the Markovian assumption may be violated.

## Results

### The model

We consider a population of leaky integrate and fire neurons driven by a common presynaptic stimulus and read-out by a decision making circuitry. To facilitate exploration both the population neurons and the decision making are stochastic. As in forced choice tasks, the decision circuitry determines a behavioral choice 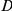 at the end of stimulus presentation, based on its monitoring of the population activity for the duration of the stimulus. We focus on binary decision making and denote the two possible behavioral choices by 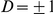. Immediately, or at some later point in time, a behavioral decision may influence whether reward is delivered to the system, but the decision may also impact the environment, i.e. influence the sequence of stimuli presented to the population neurons. Due to the last point, our framework goes beyond operant conditioning and also includes sequential decision tasks.

For the decision making circuitry itself, we use a very simple model, assuming that it only considers the number of population neurons which fire in response to the stimulus: For low population activity the likely decision is 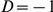, but the probability of generating the decision 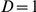 increases with the number of neurons that respond by spiking to the stimulus. Given this decision making circuitry, we present a plasticity rule for the synapses of the population neurons, which enables the system to optimize the received reward.

**Figure 2:**
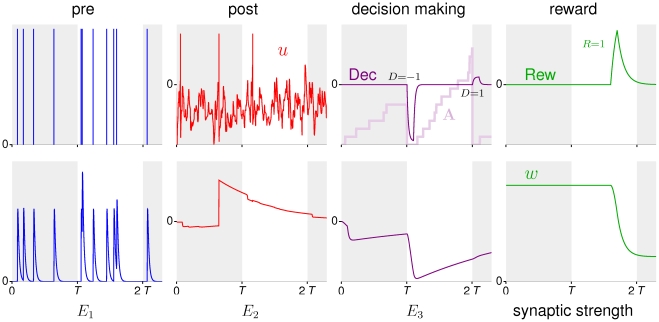
Examples for the modulatory signals and the resulting traces in the plasticity cascade of a synapse.

In presenting the plasticity rule we focus on one synapse, with synaptic strength 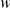, of one of the population neurons. (In the simulations, of course, the rule is applied to all synapses of all population neurons.) Let 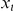 be the set of spike times representing the presynaptic spike train impinging on the synapse upto time 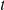. A presynaptic spike at some time 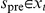 leads to a brief synaptic release with a time constant 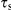 on the order of a millisecond. The postsynaptic effect of the release will however linger for a while, decaying only with the membrane time constant 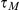 which is in the 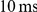 range. The first synaptic eligibility trace 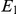 bridges the gap between the two time scales by low pass filtering (Fig. 2, column 1). It evolves as:
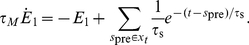
(1)


Correlations between synaptic and post-synaptic activity are captured by transcribing 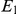 into a second trace 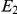 of the form
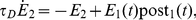
(2)see Fig. 2, column 2. The postsynaptic modulation function 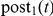 depends on the postsynaptic spike times and on the time course 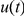 of the neuron's membrane potential. Denoting by 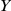 the set of postsynaptic spike times, the specific form we use for 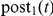 is
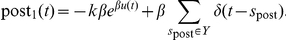
Here 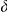 is Dirac's delta-function, 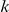 and 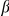 are parameters given in Methods.

As has been previously shown [14], 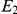 is a useful factor in plasticity rules due to the following properties:

- A small synaptic change proportional to 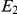 reinforces the observed neuronal response, i.e. it increases the likelihood that the neuron reproduces the observed postsynaptic spike train on a next presentation of the same stimulus.
- Conversely, a small synaptic change proportional to 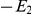 impedes the observed neuronal response. It encourages responding by a different spike train on a next presentation of the stimulus and thus facilitates exploration.

Thanks to these properties, plasticity rules where synaptic change is driven by the product of 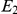 and reward have been widely used in reinforcement learning models [6], [15]–[17]. Due to 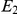, the neuronal quantities modulating plasticity in these rules are not just the pre- and post synaptic firing times but also the membrane potential 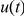. This further modulatory factor also arises in models matching STDP-experiments which measure plasticity induction by more than two spikes [18].

In our model, the time constant 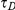 in Eq. (2) should be matched to the decision time during which stimuli are presented and we use 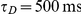. Since the match may be imperfect in reality, we denote the actual stimulus duration by the symbol 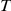. To describe the stochastic decision making in this period, we introduce the population activity variable 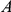 which is reset each time one decision is made and subsequently increased when a neuron spikes for the first time in response to the next presented stimulus (Fig. 2, column 3). A high (low) value of 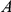 at the end of the decision period biases the next behavioral decision towards 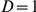 (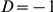). We do not model the temporal accumulation of population activity leading to 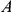 explicitly in neural terms, since this could be achieved along the lines previously suggested in [19].

Since the decision circuitry is stochastic, even for a fairly high level of population activity the behavioral decision 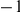 may be made by chance. In this case, by spiking, a population neuron in fact decreased the likelihood of the behavioral choice which was actually taken, whereas a neuron that stayed silent made the choice more likely. Hence, when the goal is to reinforce a behavioral decision, a sensible strategy is to reinforce a neuronal response when it is aligned with 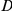 (firing for 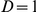, not firing for 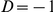) and to impede it when it is not aligned. To this end, the third eligibility trace 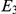 captures the interactions between single neuron activity, population activity and behavioral decision. It evolves as
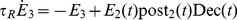
(3)where 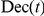 is a feedback signal, based on 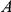 and 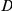, generated by the decision making circuitry and, further, 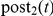 is determined by the postsynaptic activity of the neuron. Mathematically, 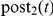 should reflect how the neuron contributed to the decision and equal 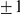 according to whether or not the neuron fired in response to the decision stimulus. The feedback signal 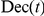 should consist of pulses generated at the times when a decision 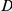 is made. The value of 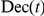 should have the same sign as the corresponding decision 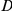 and be modulated by the population activity 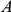 which gave rise to the decision. In particular, the magnitude of the pulse is large when 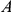 is close to the stochastic decision threshold, increasing synaptic plasticity in the cases where the decision making is still very explorative.

Since the post-stimulus value of 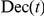 has the same sign as 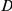, the term 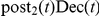 in Eq. (3) is positive when the neuronal response is aligned with the decision - otherwise it is negative. Because this term remodulates 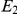 during the transcription and in view of the above characterization of 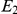, the eligibility trace 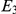 has the following property:

- A small synaptic change proportional to the post-stimulus value of 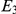 reinforces the neurons response when the response is aligned with the behavioral decision but, in the not aligned case, the response is impeded.

Since 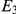 encodes the correlations between the releases of the synapse and the behavioral decision, the final stage of the cascade becomes very simple (Fig. 2, column 4). It just remodulates 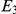 by reward to yield the synaptic change:
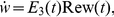
(4)Mathematically, the reward function 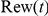 should be made up of pulses at the times when external reinforcement information becomes available, with the height of each pulse proportional to the reward received at that time.

The above description uses some mathematical idealizations which biologically are not quite realistic. We envisage that the reinforcement and decision feedback is delivered to the synapses by changes in levels of neurotransmitters such as dopamine, acetylcholine or norepinephrine [20]–[22]. Then, in contrast to the pulses assumed above, the feedback read-out by the synapses should change only quite slowly. In our simulations, this is addressed by low pass filtering the above feedback pulses when obtaining the signals 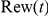 and 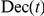. Further, we assumed above that 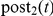 in Eq. (3) encodes whether the neuron fired in response to the decision stimulus. But it seems unrealistic, that a population neuron knows when a stimulus starts and ends. In the simulations we use low pass filtering to compute a version of 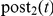 which just encodes whether the neuron spiked recently, on a time scale given by 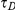 (Methods). Such a delayed feedback about postsynaptic activity could realistically be provided by calcium related signaling.

### Learning stimulus-response associations with delayed reinforcement

**Figure 3:**
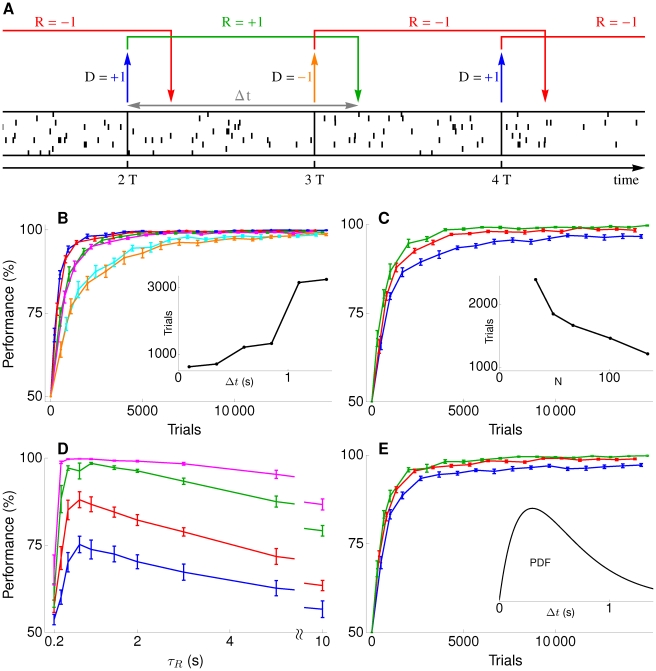
Stimulus-response association with delayed reinforcement.

To study the proposed plasticity rule, we first consider an operant conditioning like task, where for each of the stimuli presented to the network, one of the two possible behavioral decisions 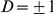 is correct. A correct decision is rewarded, whereas an incorrect one is penalized, but in both cases the delivery of reinforcement is delayed for some time. While operant conditioning with delayed reward has been widely considered in the context of temporal discounting [23], here, we are interested in a quite different issue. We do not wish to assume that little of relevance happens in the delay period between the decision and the corresponding reinforcement since this seems artificial in many real life settings. In the task we consider, during the delay period, other decisions need to be made which are themselves again subject to delayed reinforcement (Fig. 3A). Then temporal contiguity between decision and reward is no longer a proxy for causation. So the issue is not how to trade small immediate reward against a larger but later reward, but how to at all learn the association between decision and reward.

In the simulations, a stimulus is represented by a fixed spike pattern made up of 80 Poisson spike trains, each having a duration of 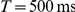 and a mean firing rate of 6 Hz. To allow for some variability, on each presentation of the stimulus, the spike times in the pattern are jittered by a zero mean Gaussian with a standard deviation of 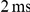. This stimulus representation is used throughout the paper. In the present task, we use 10 stimuli and, for each, one of the two possible decisions is randomly assigned as the correct one. Stimuli are presented in random order and right after the decision on one stimulus has been made, the next stimulus is presented.

Fig. 3B shows learning curves for tasks where there is a fixed delay 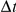 between each decision and the delivery of the reinforcement pertinent to that decision. Perfect performance is eventually approached, even for the largest value of 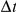 considered. For this value, 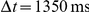, two other decisions are made in the delay period. Learning time increases in a stepwise manner when extending the delay, with a step occurring each time a further intervening decision has to be made in the delay period (Fig. 3B inset).

To demonstrate that the proposed plasticity rule addresses the spatial credit assignment problem as well, we studied learning performance as function of the number 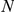 of population neurons. The results in Fig. 3C show that learning speeds up with increasing population size. In a larger population there are more synapses and the speedup indicates that the plasticity rule is capable of recruiting the additional synapses to enhance learning.

To gauge robustness, we used the same synaptic plasticity parameters for all simulations in Panels B and C. In particular 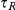 was always set to 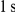 even though the actual delay 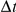 in reward delivery is varied substantially in Panel B. To further highlight robustness, Fig. 3D shows the performance for different values of 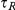 when the actual delay in reward delivery is fixed at 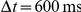.

In the above simulations the delay between decision and reward did not change from trial to trial. But the proposed plasticity rule does not rely on this for learning and also works with variable delays. This is shown in Fig. 3E, where a different, randomly chosen, delay 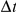 was used on each trial.

### Two armed bandit with intermittent reward

To achieve near perfect performance in the above operant conditioning task, our network had to learn to make close to deterministic decisions. Here we show that, when appropriate, the architecture can also support stochastic decision making. For this we consider a two armed bandit where one of the two targets delivers a fixed reward of 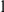 when chosen. The second choice target (which we call intermittent) will deliver a reward of 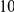 or 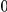 depending on whether or not the target is baited. Baiting occurs on a variable interval schedule: Once the reward of 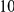 has been collected, the target becomes un-baited. It stays un-baited for between 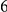 to 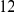 time steps (randomly chosen) and is then baited again. Once baited, the target stays in this state until it is chosen. As a consequence, always choosing the intermittent target yields an average reward equal to 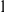. This does not improve on choosing the fixed reward target and, hence, a better policy is to pick the intermittent target less frequently.

**Figure 4:**
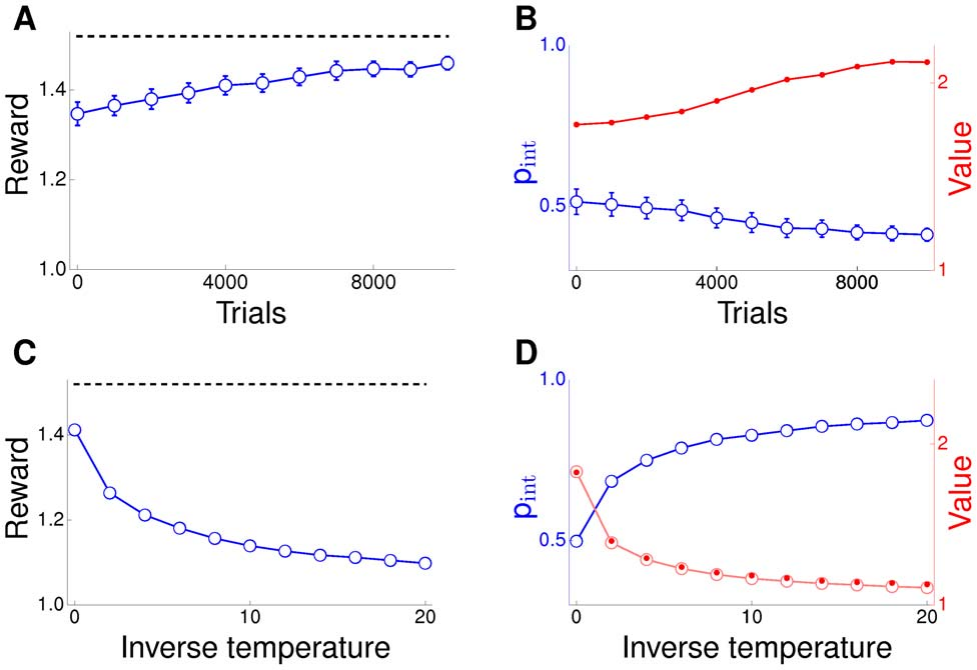
Two armed bandit with intermittent reward. Panels (A) and (B) plot the results for learning with 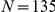 population neurons and 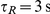. The evolution of average reward per decision is shown in (A) and compared to the reward achievable by the optimal stochastic policy (dashed line). The latter was determined by Monte Carlo simulation. The probability 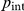 of choosing the intermittent target is shown in (B) as well as the value 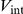, i.e the average reward obtained when choosing the intermittent target with probability 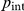. Panels (C) and (D) show the asymptotic performance of TD-learning (reached after 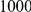 trials) for different values of the inverse temperature 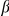. The red empty circles in panel (D) show the estimate of 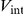 computed by the TD-algorithm. The full red circles give the exact value of 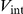 for the choice probability 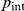 used by the TD-algorithm (blue curve).

We assume that our network does not have access to the past decisions it has made. Hence on every trial one and the same stimulus is presented to the network (with the same spike pattern statistics as in the previous subsection). The evolution of the average reward collected by the network is shown in Fig. 4A. Due to learning, average reward increases, reaching a value which is within 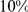 of the reward achievable by the optimal stochastic policy. The probability 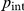 of choosing the intermittent target decreases from 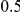 to around 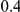 as shown in Fig. 4B. This panel also plots the evolution of the value 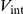 of choosing the intermittent target. The value being the expected reward collected from choosing the intermittent target assuming that the policy is to pick this target with a probability of 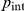.

Asymptotically 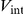 approaches a value around 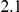. So choosing the intermittent target is much more rewarding on average than choosing the fixed target (which has a value of 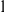). Nevertheless, the intermittent target is chosen less frequently than the fixed target. This amounts to a strong deviation from matching or melioration theory [24] which stipulates that choice frequencies adjust up to the point where the value of the two choices becomes the same - this would lead to 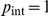 in the present task. On a task similar to ours, deviations from matching and melioration, favoring a more global optimization of reward, have also been observed in a behavioral experiment with rats [25].

Our plasticity rule, of course, does not explicitly value choices but directly adapts the choice policy to optimize overall reward. This is in contrast to temporal-difference (TD) based approaches to learning, where estimating the value of choices (or, more generally, the value of state-action pairs) is the key part of the learning procedure. Hence it is of interest to compare the above results to those obtainable with TD-learning.

The two most common strategies in TD-learning for making decisions based on the valuation of choices are 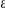-greedy and softmax. For 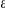-greedy the choice with the highest estimated value is taken with probability 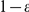, where 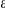 is typically a small positive parameter. This does not allow for a fine grained control of the level of stochasticity in the decision making, so we will only consider softmax here. For softmax, a decision 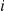 is made with a probability 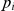 related to its value 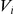 as 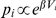. Here the positive parameter 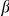, called inverse temperature, modulates the level of stochasticity in the decision making. TD-theory does not give a prescription for choosing 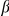 and, hence, we will consider a large range of values for the inverse temperature. The results in panels 4C and 4D plot the asymptotic performance as function of 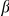. Panel 4c shows that the average reward achieved by the TD-learner decreases with increasing 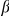. So best performance is obtained for 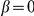, i.e. when the choice valuations estimated during learning are irrelevant. The probability 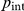 of choosing the intermittent target increases with 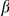, Panel 4D. The panel also shows that the estimates of 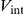 computed by the TD-algorithm are in excellent agreement to the true values of 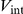 for the policy characterized by 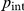. Hence, the TD-learner fails to optimize reward not because the valuation of the decisions is wrong, but it fails because softmax is a poor strategy for transforming valuations into decisions in the present task.

The root cause for the failure of TD-learning is that our decision task is not Markovian. Due to the variable interval schedule, the probability that the intermittent target is baited depends on the previous decisions made by the TD-learner. But as in the simulation on population learning, we have assumed that previous decisions are not memorized and the TD-learner is in the same state in each trial. Hence, even given the state accessible to the TD-learner, past events are nevertheless predictive of future ones because the information about the present encoded in the state is incomplete. This violates the Markovian assumption on which TD-learning theory is based. To rectify this, one needs to assume that decisions are made in view of previous decisions and outcomes. Given that the intermittent target can stay un-baited for a maximum of 12 steps, this requires a TD-learner which memorizes decisions and outcomes (reward/no reward) for the last 12 time steps. Hence, we simulated a TD-learner with the 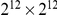 states needed to represent the task history in sufficient detail to render the decision problem Markovian. We found that the algorithm after learning (with softmax and 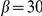) achieved an average reward of 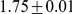 per decision. The algorithm learned to employ sophisticated policies such as not choosing the intermittent target for 8 time steps after it had delivered reward - but polling it frequently thereafter until the intermittent target again delivered reward. Obviously such policies are beyond the scope of the simple memoryless stochastic decision making considered above.

### Sequential decision making

**Figure 5:**
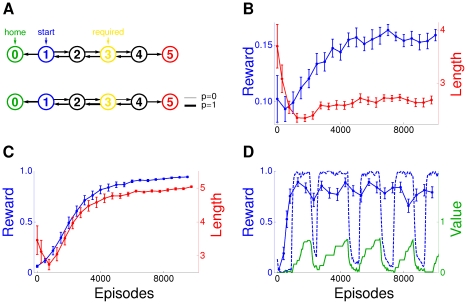
Sequential decision making. (A) Top row, sketch of the path finding task. Bottom row, example stochastic policy learned by the population when decisions are based on just the current position, arrow thickness represents probability of transition. (B) Evolution of the average reward per episode (blue) and the average number of steps per episode (red) for population learning with decisions based on current position. (C) Same as in (B), but for population learning with decisions based on the current and previous position. The above population simulations used 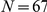 and 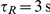. (D) TD-learning with decisions based on the current and previous position. Average reward per episode (solid blue curve) and reward per episode in a typical single run (dotted blue). For this run, the green curve shows the evolution of the value assigned by the TD-learner to making a shortcut, i.e. to the state action pair (_1_2, *left*). Error bars show 1 SEM of the mean.

We next studied population learning in a sequential decision making task, where reward delivery is contingent on making a sequence of correct decisions. For this, a simple path finding task on a linear track was used (Fig. 5A). We imagine an owner who is tired of having to take his dog for a walk and wants to teach the animal to exercise all by itself. The dog is put in front of the door (position 1 on the track), can move left or right, and may be rewarded on coming home (position 0). But since the point is to exercise the dog, reward (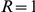) is only delivered when the dog has reached position 3 at least once while moving on the track. If the dog comes home early without visiting the required position 3, the learning episode simply ends with neither reward or punishment. The episode ends in the same way if position 5 is ever reached (the dog should not run away).

In an initial simulation, we assumed that decisions have to be made based just on the current position on the track. So the stimuli presented to the population just encode this position (using the same spike pattern statistics as in the previous tasks). Given such stimuli, our population model is faced with a non-Markovian decision problem because, the appropriateness of a decision may depend not just on the current stimulus but also on the stimuli which were previously encountered. For instance, whether one should go left or right in position 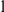 depends on whether position 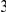 has been visited already. In fact the learning problem is even more dire. When the basis of decision making is just the current position, complete failure will result for any deterministic policy which must lead to one of the following three outcomes: (i) direct exit from position 1 to 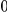, (ii) exit at position 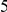, (iii) an infinite cycle. This is not to say that nothing can be learned. As the result in the bottom row of Fig. 5A shows, it is possible to increase the odds that an episode will end with reward delivery by adapting a stochastic policy. Initially the network was almost equally likely to go left or right in any position but after learning this has changed. In position 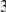 for instance left is much more likely than right, whereas, in position 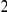, left is just a little bit more likely than right. After learning, the average number of steps per episode is lower than initially (Fig. 5B, red curve). So in terms of average reward per step taken, there is even more improvement through learning than suggested by the blue curve in Fig. 5B. In the simulations we used 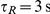. This is somewhat longer than the minimal time of 2.5 s (5 steps of 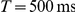 duration) needed from position 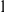 to reward delivery.

Thanks to working memory, a real dog is of course entirely capable to collect reward by simply running from position 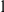 to 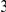 and then back to 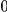. So for describing the behavior of an animal with a highly developed nervous system, the above model is woefully inadequate. Nevertheless, it may usefully account for behavior in the presence of working memory impairments. To allow for working memory, in a next set of simulations we switched to stimuli encoding not just the current but also the immediately preceeding position on the track. Of the 80 spike trains in a stimulus presented to the network, 50 were used to encode the current and 30 to encode the preceeding position (Methods). Now, learning with the proposed plasticity rule converges towards perfect performance with the reward per episode approaching 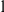 and the number of decision steps per episode approaching 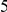 (Fig. 5C).

It is worthwhile noting, that even with a working memory reaching one step back, the decision task is non-Markovian: For instance, knowing that coming from 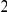 we are now in position 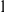 does not allow us to tell whether moving left leads to reward. For this we would need to know if we have been in position 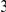, say, two steps back. Technically, when remembering the sequence of past positions, the memory depth required to make the decision problem Markovian is infinite because any finite memory can be exhausted by cycling many times between positions 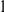 and 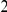. The non-Markovian nature of the task is highlighted by Fig. 5D, which shows simulation result for TD-learning. The specific algorithm used is SARSA with 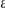-greedy decision making (see [1] and Methods). Similarly to Fig. 5C, we assumed that the states upon which the TD-learner bases decisions represents the current and the immediately preceeding position on the track. The solid blue curve in Fig. 5D, computed by averaging performance over multiple runs of the algorithm, demonstrates that TD-learning does not converge towards perfect performance. The dotted blue curve, giving results for a typical single run, shows that in fact TD-learning leads to large irregular oscillations in performance, which are averaged away in the solid curve. While optimal performance is approached initially in the single run, the algorithm is not stable and at some point performance breaks down, initiating a new cycle in the oscillation.

To understand the instability in more detail, we denote the states of the TD-learner by notation such as _2_1, meaning that coming from 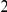 the current position is 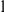. The TD-learner assigns values to state-decision pairs, which we write as e.g. (_2_1, *left*), by estimating discounted future reward. Now consider the single run of the TD-learner (dotted blue curve, Fig. 5D) after some 1500 episodes. The strategy then is close to optimal, so in most episodes when we are in state _2_1, i.e. on the inbound leg of the tour, position 3 will have previously been visited. Then *left* in _2_1 leads to immediate reward delivery, so the state-action pair (_2_1, left) has a high value. Next assume that we are on the outbound leg in state _1_2. Since the policy is close to optimal, in most episodes the next move is *right*, in order to visit position 3. But, due to exploration, the TD-learner will occasionally try the shortcut of going left in state _1_2, testing the state-action pair (_1_2, *left*). This leads to state _2_1 and then most likely to the high value decision *left*, terminating the episode without reward because the shortcut was taken. But the TD-learner updates the value of the tested state-action pair (_1_2, *left*) based not on the failure at the very end of the episode but based on the value of the subsequent state-action pair, in this case (_2_1, *left*). As noted above, the latter pair has high value, so the update increases the value of the shortcut (_1_2, *left*) even-though the shortcut resulted in failure (green curve in Fig. 5D). This happens most of the times when the shortcut is tested for exploration, leading to further increases in the green curve, upto the point where the value of (_1_2, *left*) is so high that making a shortcut becomes the dominant policy. This causes the observed breakdown in performance. In summary, a central idea in temporal difference learning is to handle non-immediate reward by back-propagating it in time via the valuations of intermediate state-decision pairs. This is mathematically justified in the Markovian case, but may lead to unexpected results for general sequential decision making tasks.

## Discussion

We have presented a model of reinforcement learning in a population of spiking neurons read out by a decision making circuitry where plasticity induction is controlled by a cascade of synaptic memory traces. In each synapse of the population neurons, the presynaptic trace is in stages remodulated by somatic feedback, by feedback about the behavioral decision making and by an external reward signal before being consolidated into a persistent change of the synaptic strength. Simulation results show that this leads to robust learning performance in a variety of reinforcement tasks.

Our model builds on, but goes beyond, the classical STDP findings [2],[3],[26]. On the neuronal level, we assume that plasticity does not only depend on the timings in a pre- and postsynaptic spike pair but that there is a further modulation by postsynaptic subthreshold activity. Such a modulation also arises when modeling the plasticity findings obtained when the standard STDP-protocol is extended to allow multi spike interactions [18]. For reinforcement learning, plasticity cannot be blind to activity-related downstream information. This matches with experimental observations revealing that the polarity and magnitude of STDP can in fact be regulated by neuromodulators such as dopamine, acetylcholine or noradrenaline which may even revert the sign of the synaptic change [10], [21], [22], e.g. by entering after the mGluR signaling pathways [27]–[29]. Some recent research has further highlighted astrocytes as local communication elements which are capable of modulating synaptic plasticity [30], [31]. Research on synaptic tagging has revealed the astonishingly large time span during which the consolidation of early-LTP into long lasting synaptic change can be dependent on behavioral reinforcement [32], [33]. The present work provides a phenomenological model showing how the multi-stage processes observed in the induction of long-term synaptic plasticity can be bound into a functional whole.

Previous modeling of population learning has already considered the modulation of plasticity by feedback from the decision circuitry [16], [34]. However, in these works the cascade was shortcut, with decision and reward feedback interacting directly in the modulation of plasticity. As a consequence the previous plasticity rule was capable of handling delays between decision and reward feedback only when these where very small, namely a fraction of typical stimulus duration. The present rule achieves a far more general solution to the temporal credit assignment problem by using a further stage in the synaptic cascade to decouple decision from reward feedback. Further, the rule is now based directly on optimizing the average reward rate (Text S1) and not just, as previously, a related objective function. This puts the present approach squarely into the field of policy gradient methods [35]–[37]. Within this field, our main contribution is to show how the spatial credit assignment problem of distributing the learning between the population neurons can be solved in a biophysically plausible way. As the results in the section on learning stimulus-response association demonstrate, our plasticity rule leads to a learning performance which scales well to large population sizes (a more detailed scaling analysis has been given in [34]). This is in contrast to the straightforward policy gradient approach of treating the neurons as independent agents which results in a rapid deterioration of learning performance with increasing population size [16].

Crucially in our population model neurons need to cooperate in order to receive reward and hence during learning a difficult spatial credit assignment problem arises. The appropriateness of any single neuron response cannot be determined without taking the responses of the other neurons into account and hence synapses in different neurons need to co-adapt in optimizing reward. This is in contrast to previous work [38] modeling a biofeedback experiment in monkeys [39] where reward delivery was contingent on the firings of a single target neuron. In the model [38] background activity was high, so that reinforcement could be increased by simply strengthening the synapses of the target neuron without any need for coordinated adaptation by the other neurons in the system.

Some parameters in our plasticity scheme are related to properties of the learning task. For instance the time constant 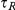 in the last stage of the cascade represents a guess at the typical delay between decision and reinforcement. Our simulation results indicate that learning is not overly sensitive to the choice of the synaptic parameters (see e.g. Fig. 3D). Nevertheless, learning does of course deteriorate once the mismatch between synaptic and actual task parameters becomes too large. An intriguing possibility for further increasing robustness could be an inhomogeneous population of neurons. After all, a key point in population coding is to provide redundancy [40], [41]. This is borne out by findings in [16] where, with increasing population size, decision performance improves but the correlation between single neuron performance and decision decreases. Hence it is of interest to study learning when different population neurons have different synaptic parameters. Then the neurons with parameters best matched to the task at hand, are expected to learn best. Thanks to their resulting correlated activity, they should be able to carry the population decision because the contributions from the badly learning mismatched neurons should be uncorrelated and thus tend to cancel. Unfortunately, meaningfully testing whether neuronal variability increases robustness in this manner, requires the simulation of population sizes which are an order of magnitude larger than what is currently within our computational reach.

With regard to the temporal credit assignment problem, we think it is important to note that delayed interaction between decision making and reward delivery can arise in diverse manners:

- *Delays in causation.* Sometimes it just takes a while till the effect of decisions and actions becomes apparent - as when taking a pill against headache.
- *Incomplete information.* The stimulus on which the decision is based does not encode all of the decision relevant information. Then previous stimuli and decisions can be of importance to the current decision because they induce a bias on the missing information. A case in point is the two armed bandit task, where previous decisions influence the odds that the intermittent target is baited. If, in contrast, the decision stimulus where to encode whether or not the intermittent target is baited, optimal decision making would be possible based just on the current stimulus.
- *Moving towards a rewarding state.* Appropriate decisions or actions are needed to navigate through a set of intermediate non-rewarding states towards a rewarding goal - as when first going to the kitchen, then opening the fridge in order to finally get a beer. In contrast, for the sequential decision making task we considered above, reward is not just contingent on reaching the home state but also on the path taken.

Policy gradient methods work in all of the above settings. Of course, missing information can be detrimental to the performance which is achievable at all. But, given this constraint, policy gradient methods will nevertheless optimize the performance. Temporal difference (TD) methods, however, by design handle only problems of type *iii*. In the first two cases TD-learning only applies when the state which serves as basis for the decision making represents the recent task history to the extent that the problem becomes Markovian. Formally, this maps the first two kinds of delays onto the third kind.

Representing the recent task history is what working memory is good for - and working memory is well known to enter into decision making as in delayed match to sample tasks. On the other hand, transforming a non-Markovian into a Markovian decision problem can pose daunting demands on the working memory capacity needed to adequately represent the states in the TD-algorithm. With insufficient working memory the algorithm can fail in two distinct ways. The estimates for the value of some state-action pairs may be wrong (as demonstrated in the sequential decision making task), or, even when the estimates are correct, preferentially choosing the available action with highest estimated value may lead to a suboptimal policy (as in the two armed bandit).

Policy gradient methods such as our population learning rule seem attractive as basic biological models of reinforcement learning because they work in a very general setting. Arguably, this generality is also a drawback. Precisely because the Markovian property is restrictive, exploiting it in the cases where it does apply, can substantially speed up learning. Hence, it is of interest that policy gradient methods can easily be combined with TD-state valuations in the framework of actor-critic methods. This amounts to simply replacing the direct reward signal in the policy gradient plasticity rule with a signal generated by the TD-valuation circuitry. The TD-signal can either be the estimated value of the current state [42] or the value prediction error [15]. Combining policy gradient with TD-valuations in this way, again brings about the Markovian restriction. Hence, if reinforcement learning is to be both robust and fast, issues of metaplasticity arise: How does brain learn how to learn when?

## Methods

### Population neurons

The model neurons in our population are escape noise neurons [14], i.e. leaky integrate and fire neurons where action potentials are generated with an instantaneous firing rate which depends on the membrane potential. Focusing on one of the population neurons, we denote by 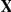 its input which is a spike pattern made up of 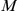 spike trains 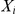 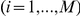. Each 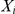 is a list of the input spike times in afferent 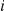. We use the symbol 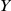 to refer to the postsynaptic spike train produced by the neuron, 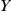 is also a list of spike times. If the neuron, with synaptic vector 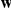, produces the output 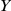 in response to 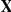, its membrane potential is determined by
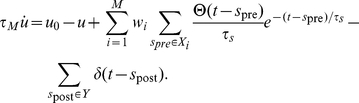
(5)Here 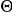 is the unit step function and, further, 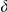 is Dirac's delta function, leading to immediate hyperpolarization after a postsynaptic spike. For the resting potential, denoted above by 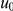, we use 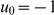 (arbitrary units). Further, 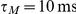 is used for the membrane time constant and 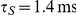 for the synaptic time constant.

By integrating the differential equation, the membrane potential can be written in spike response form as
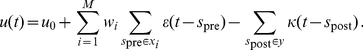
(6)The postsynaptic kernel 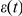 and the reset kernel 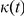 vanish for 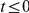. For 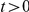 they are given by
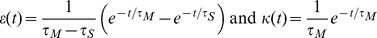
Note that the first eligibility trace 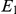 of synapse 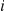 can be expressed in terms of the postsynaptic kernel as 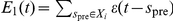.

Action potential generation is controlled by an instantaneous firing rate 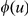 which increases with the membrane potential. So, at each point 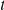 in time, the neuron fires with probability 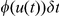 where 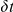 represents an infinitesimal time window (we use 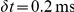 in the simulations). Our firing rate function is
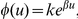
with 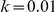 and 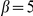. (In the limit of 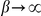 one would recover a deterministic neuron with a spiking threshold 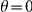.)

As shown in [14], the probability density, 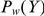, that the neuron actually produces the output spike train 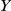 in response to the stimulus 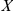 during a decision period lasting from 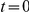 to 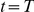 satisfies:
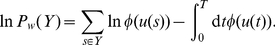
(7)The derivative of 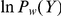 with respect to the strength of synapse 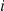 is known as characteristic eligibility in reinforcement learning [35]. For our choice of the firing rate function one obtains
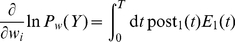
(8)where 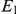 is the first eligibility trace of the synapse (Eq. 1) and 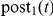 the postsynaptic signal of the neuron given right below Eq. (2). Note that (8) is similar to our second eligibility trace 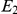, see Eq. (2), except that we have replaced the integration over the decision period by low pass filtering with a time constant matched to the stimulus duration. The reason for this is that it seems un-biological to assume that the synapses of the population neurons know when decision periods start and end.

### Architecture and decision making

We use the superscript 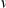, running from 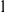 to 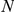, to index the population neurons. For instance, 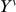 is the postsynaptic spike train produced by neuron 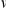 in response to its input spike pattern 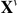. As suggested by the notation, the population neurons have different inputs, but their inputs are highly correlated because the neurons are randomly connected to a common input layer which present the stimulus to the network. In particular, we assume that each population neuron synapses onto a site in the input layer with probability 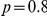, leading to many shared input spike trains between the neurons.

The population response is read out by the decision making circuitry based on a spike/no-spike code. For notational convenience we introduce the coding function 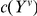, with 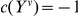, if the there is no spike in the postsynaptic response 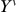, otherwise, if neuron 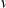 produce at least one spike in response to the stimulus, 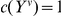. In term of this coding function the population activity 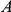 being read out by the decision making circuitry can be written as:
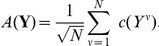
Using this activity reading, the behavioral decision 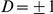 is made probabilistically, the likelihood 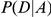 of producing the decision is given by the logistic function
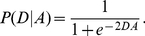
(9)Note that due to the 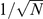 normalization in the definition of 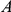, the magnitude of 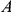 can be as large as 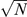. This is why, decisions based on the activity of a large population can be close to deterministic, despite of the noisy decision making circuitry.

### Feedback signals and the postsynaptic trace

We start with the reward feedback 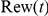, modulating synaptic plasticity in Eq. (4). This feedback is encoded by means of a concentration variable 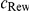, representing ambient levels of a neurotransmitter, e.g. dopamine. In the absence of reward information, the value of 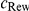 approaches a homeostatic level 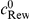 with a time constant 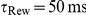. For any point in time 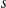 when external reward information 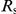 is available, this reinforcement leads to a change in the production rate of the neurotransmitter. The change is proportional to 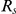 and lasts for 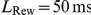. So up to the point in time 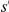 when further reinforcement becomes available, the concentration variable evolves as:
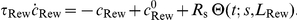
Here the step function 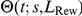 equals 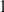 if 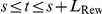, otherwise the function value is zero. The reward feedback read-out at a synapse is determined by the deviation of the current neurotransmitter level 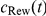 from its homeostatic value and equals
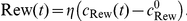
Here the parameter 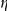 is the positive learning rate which, for notational convenience, we absorb into the reward signal.

The decision feedback 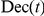 used in Eq. (3) is encoded in the concentration 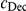 of a second neurotransmitter. As for reward feedback, this is achieved by a temporary change in the production rate of the encoding neurotransmitter. For describing 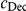, we assume a stimulus that ended at time 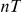, evoking the population activity 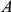 and behavioral decision 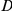. As shown in Text S1, the value of 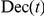 should then be determined by the derivative of 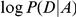 with respect to 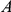 and, in view of Eq. (9), this derivative is simply 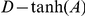. Hence we use
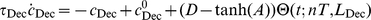
for the temporal evolution of 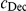. Parameter values in the simulations are 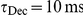 and 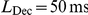. The above equation holds up to time 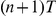 when the subsequent stimulus presentation ends, at which point the decision variables 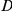 and 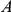 are replaced by their values for the latter stimulus. The decision feedback 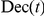 is simply
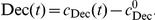



For the postsynaptic trace 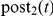 in Eq. (3), we assume a concentration variable 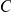 which reflects the spiking of the neuron. Each time there is a postsynaptic spike, 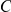 is set to 1; at other times, 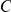 decays as 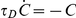. The value of 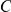 should reflect whether or not the neuron spiked in response to the decision stimulus. So, as for the eligibility trace 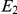 (see Eq. 2), the relevant time scale is the decision period and this is why the same time constant 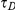 is used in both cases. The trace 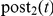 is obtained as
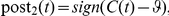
comparing 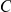 to an appropriate threshold 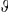. In the simulation we use 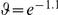. For the reasoning behind this choice, consider a stimulus ending at time 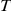 of duration 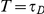. The value of 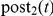 at time 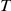 will accurately reflect whether or not the decision stimulus elicited a postsynaptic spike, if we choose 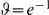. But since decision feedback is not instantaneous, the value of 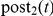 is mainly read-out at times later than 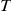. This is why the smaller value 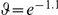 seemed a somewhat better choice.

### TD-learning

For TD-learning we used the SARSA control algorithm [1] which estimates the values of state-action pairs 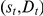. At each point in time, the value estimates 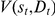 are updated according to
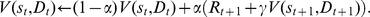
Here 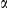 and 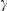 have values between 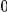 and 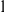. The parameter 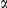 is similar to a learning rate and 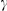 controls the temporal discounting. The above update is done after every transition from a nonterminal state 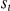. If 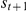 is terminal, then 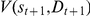 is defined as zero. When in state 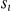, the next action 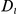 is chosen using either 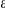-greedy or softmax. In both cases only the values 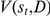 pertinent to the current state enter into the decision making.

For memoryless TD-learning in the two armed bandit we used 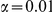 and 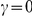. A positive discount factor would not qualitatively change the result. For each of 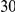 runs per chosen value of 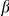, we simulated 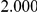 trials. After 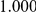 trials learning had converged and the reported asymptotic quantities are the average over the next 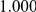 trials. For learning with memory we used 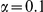, 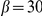 and 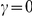.

For the sequential decision making task decision selection used 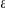-greedy with 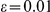. The discount factor was set to 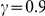 and the step-size parameter to 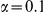.

With regard to the failure of TD-learning in the sequential decision making task, we note that there are also eligibility trace based versions, SARSA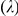, of the algorithm with the above version corresponding to 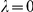. For 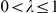, the value update takes into account not just the next state-action pair but the value of all subsequent state-action pairs. Importantly, for the special case 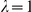 the subsequent values occurring in the update cancel, and the value update is in effect driven directly by the reward signal [1]. So SARSA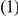 is just a complicated way of doing basic Monte Carlo estimation of the values. It hence does not assume that the process is Markovian and SARSA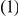 does reliably converge towards optimal performance in our task. For 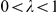 the procedure interpolates between the two extremes 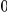 and 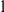. Consequently the valuation of some state-action pairs (e.g. the shortcut _1_2, *left*) will then be wrong but the error will be smaller than for 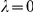. If action selection is based on softmax the incorrect valuation will nevertheless be detrimental to decision making. However, this need not always be the case for 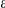-greedy, due to the thresholding inherent in this decision procedure. In particular, there is a positive critical value for 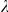 (which depends mainly on the discount factor 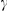) above which the valuation error will no longer affect the decision making. In this parameter regime, SARSA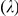 will reliably learn the optimal policy (upto the exploration determined by 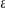).

### Miscellaneous simulation details

In all the simulations initial values for the synaptic strength were picked from a Gaussian distribution with mean zero and standard deviation equal to 4, independently for each afferent and each neuron.

A learning rate of 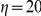 was used in all simulations, except for the 2-armed bandit task where 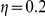 was used.

In the sequential decision making task with working memory, the population is presented stimuli encoding not just the current but also the immediately preceeding position. For this, each location on the track is assigned to a fixed spike pattern made up of 50 spike trains representing the location in the case that it is the current position and, further, to a second spike pattern with 30 spike trains for the case that it is the immediately preceeding position. The stimulus for the network is then obtained by concatenating the 50 spike trains corresponding to the current position with the 30 spike trains for the preceeding position.

The curves showing the evolution of performance were obtained by calculating an exponentially weighted moving average in each run and then averaging over multiple runs. For the sequential decision making task reward per episode was considered and the smoothing factor in the exponentially weighted moving average was 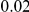. In the other task, where performance per trial was considered, the smoothing factor was 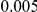. For each run a new set of initial synaptic strength and a new set of stimuli was generated. The number of runs was 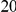, except in the two armed bandit where we averaged over 40 runs.

## Supporting Information

Text S1 We show how the plasticity rule presented in the main text is based on a gradient ascent procedure maximizing the average reward rate.
(PDF)
