# Supplementary material for: Spatio-Temporal Credit Assignment in Neuronal Population Learning
Source: PLoS Comput Biol. 2011 Jun 30;7(6):e1002092. doi: 10.1371/journal.pcbi.1002092 (PMC3127803; doi:10.1371/journal.pcbi.1002092)
Supplement: Text S1 — We show how the plasticity rule presented in the main text is based on a gradient ascent procedure maximizing the average reward rate. (PDF) [file pcbi.1002092.s001.pdf]

# Spatio-temporal credit assignment in neuronal population learning. Text S1: Relating the plasticity rule to a gradient ascent procedure

Johannes Friedrich[1], Robert Urbanczik[2], Walter Senn[3,*]
**1,2,3 Department of Physiology, University of Bern, Bühlplatz 5, CH-3012 Bern, Switzerland**
**$*$ E-mail: senn@pyl.unibe.ch**

We show how the plasticity rule presented in the main text is based on a gradient ascent procedure maximizing the average reward rate. This Supplementary Material is organized as follows: First, a formula is derived for the gradient, with respect to synaptic strength of the population neurons, for the probability of taking a behavioral decision. We next summarize Baxter and Bartlett's framework (Ref. [1] main text) for learning in partially observable Markov decision processes (POMDP's). Finally, we show how our population gradient leads to a learning rule for POMDP's and how this procedure, formulated in discrete time, transforms to the simplified online plasticity rule presented in the main text.

## 1 Gradient for the behavioral decision

Let $\mathbf{X}$ be the spike pattern presented to the population neurons and $\mathbf{W}$ the matrix of their synaptic strength. The probability $P_{\mathbf{W}}(D|\mathbf{X})$ of responding with decision $D$ to the stimulus is

$$P_{\mathbf{W}}(D|\mathbf{X}) = \int \mathrm{d}\mathbf{Y}\, P(D|A(\mathbf{Y})) \prod_{\nu=1}^{N} P_{\mathbf{W}^\nu}(Y^\nu|\mathbf{X}^\nu)\,.$$

Here, the conditional probability of the decision, $P(D|A)$, is given by Eq. (9, main text), the definition of the activity $A(\mathbf{Y})$ is given just above Eq. (9, main text). The probability $P_{\mathbf{W}^\nu}(Y^\nu|\mathbf{X}^\nu)$ that neuron $\nu$ produces the postsynaptic spike train $Y^\nu$ in response to its version $\mathbf{X}^\nu$ of the stimulus is obtained by applying Eq. (7, main text) to neuron $\nu$.

We will only need the gradient of $P_{\mathbf{W}}(D|X)$ for a single stimulus. So, ignoring the dependence on $X$, we write

$$P_{\mathbf{W}}(D) = \int \mathrm{d}\mathbf{Y}\, P(D|A(\mathbf{Y})) \prod_{\nu=1}^{N} P_{\mathbf{W}^\nu}(Y^\nu)\,. \quad (1)$$

To lighten the notation further, we focus on calculating the gradient of $P_{\mathbf{W}}(D)$ only with respect to the strength of one of the synapses (the expressions for the other synapses being entirely analogous). Let $w$ denote the strength of the first synapse of the first population neuron and let $Y = Y^1$ the postsynaptic spike train produced by this neuron. To isolate the contribution of the first neuron we decompose the activity $A(\mathbf{Y})$ as

$$A(\mathbf{Y}) = \tfrac{1}{\sqrt{N}} c(Y) + A^{\setminus}(Y^2, \ldots, Y^N) \quad \text{with } A^{\setminus} = \frac{1}{\sqrt{N}} \sum_{\nu=2}^{N} c(Y^\nu)\,.$$

As random variables, $Y$ and $A^{\setminus}$ are independent, since the probability density on the postsynaptic spike trains $\mathbf{Y}$ in Eq. (1) is given by a product. Further the density on $Y^2, \ldots, Y^N$ induces a density $\mu$ on $A^{\setminus}$, crucially $\mu$ does not depend on $w$. With this notation, we can rewrite (1) as

$$P_w(D) = \int \mathrm{d}Y \mathrm{d}A^{\setminus} \mu(A^{\setminus})\; P\left(D \,|\, \tfrac{c(Y)}{\sqrt{N}} + A^{\setminus}\right)\; P_w(Y)\,.$$

We now set

$$\begin{aligned} \mathrm{S}P(D|A^{\setminus}) &= \tfrac{1}{2}\left(P(D\,|\,\tfrac{1}{\sqrt{N}}+A^{\setminus})+P(D\,|\,\tfrac{-1}{\sqrt{N}}+A^{\setminus})\right)\\ \mathrm{D}P(D|A^{\setminus}) &= \tfrac{1}{2}\left(P(D\,|\,\tfrac{1}{\sqrt{N}}+A^{\setminus})-P(D\,|\,\tfrac{-1}{\sqrt{N}}+A^{\setminus})\right) \end{aligned} \tag{2}$$

and have

$$P\left(D\,|\,\tfrac{c(Y)}{\sqrt{N}}+A^{\setminus}\right)=\mathrm{S}P(D|A^{\setminus})+\mathrm{D}P(D|A^{\setminus})c(Y)\,.$$

Plugging this into the above expression for $P_w(D)$ we obtain

$$P_w(D)=\int\!\mathrm{d}A^{\setminus}\mu(A^{\setminus})\ \mathrm{S}P\left(D|A^{\setminus}\right)+\int\!\mathrm{d}Y\mathrm{d}A^{\setminus}\mu(A^{\setminus})\ \mathrm{D}P\left(D|A^{\setminus}\right)\ c(Y)\ P_w(Y)\,.$$

Now, since the first integral does not depend on $w$, we have

$$\tfrac{\partial}{\partial w}P_w(D)=\int\!\mathrm{d}Y\mathrm{d}A^{\setminus}\mu(A^{\setminus})\ \mathrm{D}P(D|A^{\setminus})\,c(Y)\ \tfrac{\partial}{\partial w}P_w(Y)\,.$$

To bring this result into a form which is usable in the Monte Carlo sampling procedure below, we first rewrite it as

$$\begin{aligned} \tfrac{\partial}{\partial w}P_w(D) &= \int\!\mathrm{d}Y\mathrm{d}A^{\setminus}\mu(A^{\setminus})\ \mathrm{D}P(D|A^{\setminus})\,c(Y)\tfrac{\partial}{\partial w}P_w(Y)\\ &= \int\!\mathrm{d}Y\mathrm{d}A^{\setminus}\mu(A^{\setminus})\ P(D|A)\frac{\mathrm{D}P\big(D|A^{\setminus}\big)}{P(D|A)}\,c(Y)P_w(Y)\,\frac{\frac{\partial}{\partial w}P_w(Y)}{P_w(Y)}\\ &= \int\!\mathrm{d}Y\mathrm{d}A^{\setminus}\mu(A^{\setminus})P_w(Y)P(D|A)\ \frac{\mathrm{D}P\big(D|A^{\setminus}\big)}{P(D|A)}\,c(Y)\tfrac{\partial}{\partial w}\ln P_w(Y)\,, \end{aligned}$$

where from the second line on we have used $A$ as shorthand for $A=\frac{1}{\sqrt{N}}c(Y)+A^{\setminus}$. The product of densities the third line, $\mu(A^{\setminus})P_w(Y)P(D|A)$, is just the joint density, $\mu(A^{\setminus})P_w(Y)P(D|A)=P_w(D,A^{\setminus},Y)$. For the Monte Carlo procedure we decompose the joint density as $P_w(D,A^{\setminus},Y)=P_w(D)P_w(A^{\setminus},Y|D)$ to obtain our final expression for the gradient

$$\tfrac{\partial}{\partial w}P_w(D)=P_w(D)\int\!\mathrm{d}Y\mathrm{d}A^{\setminus}P_w(A^{\setminus},Y|D)\ \frac{\mathrm{D}P\big(D|A^{\setminus}\big)}{P(D|A)}\,c(Y)\tfrac{\partial}{\partial w}\ln P_w(Y) \tag{3}$$

# 2 Policy gradient learning in a POMPD

A partially observable Markov decision problem involves a finite number of states which we shall denote by lower case bold symbols such as $\mathbf{i}$ or $\mathbf{j}$. Each state is associated with a reward value $R(\mathbf{i})$ which may depend deterministically or stochastically on $\mathbf{i}$. The behavior of the learning agent iteratively generates a discrete time Markov process as follows. Assuming the process is in state $\mathbf{i}$ at discrete time step $n-1$, then:

- The agent makes an observation $\mathbf{X}(\mathbf{i})$, which may be partial and noisy, so it may be impossible to uniquely identify the underlying state $\mathbf{i}$ based on $\mathbf{X}(\mathbf{i})$. As the notation suggests, $\mathbf{X}(\mathbf{i})$ corresponds to the stimulus presented to our decision network.
- Based on the observation, the agent generates a control, according to an adaptable stochastic policy. In our parlance, a control corresponds to a decision D, so the agent is described by $P_w(D|\mathbf{X}(\mathbf{i}))$, where $w$ is an adaptable parameter.

- On the next time step $n$, the process transitions to state $\mathbf{j}$ with probability $p_{\mathbf{i}\to\mathbf{j}}(D)$, where $D$ is the decision just made by the agent.
- The agent receives reward $R(\mathbf{j})$.

As a consequence of the decisions made, the agent thus receives a sequence of rewards $R(\mathbf{j}_n)$, and the goal of the agent is to maximize the long term average reward rate $r(w)$. Formally

$$r(w) = \lim_{N\to\infty} \frac{1}{N} \left\langle \sum_{n=1}^{N} R(\mathbf{j}_n) \right\rangle$$

where the angle brackets denote the expectation over the stochastic process.

Before presenting the gradient rule for maximizing $r(w)$, let us show how this framework applies to the learning problems studied in the main text. As example we consider the stimulus response association task when reward is delayed by $\Delta t = 650\,\text{ms}$, i.e. a bit longer than the 500 ms duration of a single stimulus. Then, at the onset of the presentation of $n$-th stimulus, the Markovian state $\mathbf{j}_n$ comprises the following three elements:

$\mathbf{j}_n^S$: The pure version of the $n$-th stimulus.

$\mathbf{j}_n^{c_1}$: A flag which is $\pm 1$ according to whether the decision in response to stimulus $n-1$ was correct.

$\mathbf{j}_n^{c_2}$: A $\pm 1$ flag according to whether the response to stimulus $n-2$ was correct.

The partial observation $\mathbf{X}(\mathbf{j}_n)$ actually presented to the network is a jittered version of the stimulus $\mathbf{j}_n^S$. The reward $R(\mathbf{j}_n)$, delivered 150 ms into the presentation of stimulus $n$, is the value of $\mathbf{j}_n^{c_2}$. Once the network has responded to stimulus $n$, the flag $\mathbf{j}_{n+1}^{c_1}$ in the next state $\mathbf{j}_{n+1}$ is set according to the correctness of this response. Further, for $\mathbf{j}_{n+1}^S$ a next stimulus is picked at random and, finally, the $\mathbf{j}_{n+1}^{c_2}$ flag is set from $\mathbf{j}_n$ using $\mathbf{j}_{n+1}^{c_2} = \mathbf{j}_n^{c_1}$.

Baxter and Bartlett consider the following eligibility trace computed while sampling the decision process:

$$e_{n+1} = (1-\gamma)e_n + \frac{\frac{\partial}{\partial w} P_w(D_n|\mathbf{X}(\mathbf{j}_n))}{P_w(D_n|\mathbf{X}(\mathbf{j}_n))} \tag{4}$$

Here $0 < \gamma < 1$ is a discount factor and $D_n$ is the decision actually made in the $n$-th time step. They next introduce the reward weighted average of $e_n$,

$$g_n = \frac{1}{n} \sum_{m=1}^{n} R(\mathbf{j}_m) e_m$$

and relate $g_n$ to the gradient of a suitable objective function which we denote by $r_\gamma(w)$. In more detail, Baxter and Bartlett show that, under mild regularity conditions on the decision process

$$\lim_{n\to\infty} g_n = \tfrac{\partial}{\partial w} r_\gamma(w) \quad \text{with probability 1.}$$

Of course, what we actually want to maximize is the average reward rate $r(w)$ and the relationship to the objective function is

$$\lim_{\gamma\to 0} r_\gamma(w) = r(w)\,.$$

But to assure that $g_n$ has finite variance, we need to use a positive value of $\gamma$. So for the proposed procedure there is a bias-variance tradeoff in choosing the discount factor $\gamma$. In a companion paper

(Ref. [2] main text), Baxter and Bartlett show that the above result leads to the following stochastic gradient procedure for the online adaption of $w$:

$$w_n - w_{n-1} = \eta_n R(j_n) e_n$$

with positive learning rates $\eta_n$. Technically, to assure convergence, one has to assume that $\eta_n$ is not fixed in time but that it decays to 0 according to a suitable schedule. For biological systems this solution to the stability-plasticity dilemma seems unrealistic and, below, we shall stick to a fixed learning rate.

# 3 Population learning for POMDP's

To arrive at a first version of our population learning procedure we simply plug Eq. (3) in to Eq. (4)

$$e_{n+1} = (1-\gamma)e_n + \int \mathrm{d}Y \mathrm{d}A^{\setminus} P_w(A^{\setminus}, Y|D_n, \mathbf{X}_n)\ \tfrac{\mathrm{D}P\left(D_n|A^{\setminus}\right)}{P(D_n|A)}\, c(Y) \tfrac{\partial}{\partial w} \ln P_w(Y|\mathbf{X}_n)\,. \tag{5}$$

Here $\mathbf{X}_n$ is the stimulus presented at the $n$-th time step and in contrast to Eq. (3) we now make the stimulus dependence explicit in the notation. The fixed learning rate update simply is

$$w_n - w_{n-1} = \eta R_n e_n\,.$$

As it stands the above procedure is an unhappy compromise. The averaging over stimuli, decisions and rewards is achieved by Monte-Carlo sampling during the evolution of the decision process, whereas the averaging over the variables $A^{\setminus}$ and $Y$, internal to the decision network, is done separately for each time step in Eq. (5). Since, conditioned on the decisions $D_n$, the evolution of the process is independent of the internal variables, it is more natural to also leave the averaging over $A^{\setminus}$ and $Y$ to the Monte-Carlo procedure. This amounts to simply using

$$e_{n+1} = (1-\gamma)e_n + \tfrac{\mathrm{D}P\left(D_n|A^{\setminus}_n\right)}{P(D_n|A_n)}\, c(Y_n) \tfrac{\partial}{\partial w} \ln P_w(Y_n|\mathbf{X}_n)\,. \tag{6}$$

instead of Eq. (5). In first instance, the sampling prescription for the decision process now is to pick $D_n$ from $P_w(D_n|\mathbf{X}_n)$ and then pick $A^{\setminus}_n$ as well as $Y_n$ from $P_w(A^{\setminus}_n, Y_n|D_n, \mathbf{X}_n)$. But this just amounts to sampling the joint density $P_w(D_n, A^{\setminus}_n, Y_n|\mathbf{X}_n)$. The natural way to sample this joint density is to have the population generate spike trains in response to the stimulus, use this to calculate $A^{\setminus}_n$ as well as $A_n = A^{\setminus}_n + c(y)/\sqrt{N}$, and then sample $P(D_n|A_n)$.

The expression for $e_n$ can be simplified by assuming that the population size $N$ is large. We can then replace the finite difference in $\mathrm{D}P\left(D_n|A^{\setminus}_n\right)$, see Eq. 2, by a differential obtaining

$$\begin{aligned}\mathrm{D}P\left(D_n|A^{\setminus}_n\right) &= \tfrac{1}{\sqrt{N}} \tfrac{\partial}{\partial A^{\setminus}_n} P\left(D_n|A^{\setminus}_n\right) + \mathcal{O}(1/N)\\ &= \tfrac{1}{\sqrt{N}} \tfrac{\partial}{\partial A_n} P\left(D_n|A_n\right) + \mathcal{O}(1/N)\end{aligned}$$

where in the last line we have used that the difference between $A^{\setminus}_n$ and $A_n$ is $\mathcal{O}(1/\sqrt{N})$. We use this for simplifying (6) to

$$e_{n+1} = (1-\gamma)e_n + \mathcal{O}(1/N) + \left(\tfrac{\partial}{\partial w} \ln P_w(Y_n|\mathbf{X}_n)\right) c(Y_n) \tfrac{1}{\sqrt{N}} \tfrac{\partial}{\partial A_n} \ln P(D_n|A_n)\ . \tag{7}$$

We can now compare this to the last eligibility trace $E_3$ of the online cascade proposed in the main text

$$\tau_R \dot{E}_3 = -E_3 + E_2(t)\mathrm{post}_2(t)\,\mathrm{Dec}(t)$$

and observe the following correspondences between the increment in $E_3$ and in Eq. (7). As mentioned in Methods, $E_2$ is a low pass filtering approximation to $\frac{\partial}{\partial w} \ln P_w(Y_n|\mathbf{X}_n)$ and $\text{post}_2(t)$ is the continuous encoding of $c(Y_n)$. For the specific decision circuitry used, $\frac{\partial}{\partial A_n} \ln P(D_n|A_n) = D_n - \tanh(A_n)$. The latter term provides the modulation of the decision feedback $\text{Dec}(t)$ given in Methods. For the online procedure, the $1/\sqrt{N}$ factor in (7) is absorbed into the learning rate. Finally the discount factor $\gamma$ corresponds to the ratio of stimulus duration $T$ to $\tau_R$.

# References


1. Baxter J, Bartlett P (2001) Infinite-horizon policy-gradient estimation. J Artif Intell Res 15: 319-350.

2. Baxter J, Bartlett P, Weaver L (2001) Experiments with infinite-horizon, policy-gradient estimation. J Artif Intell Res 15: 351-381.
